# Effect of Kidney Dysfunction on Cerebral Cortical Thinning in Elderly Population

**DOI:** 10.1038/s41598-017-02537-y

**Published:** 2017-05-24

**Authors:** Chih-Hao Chen, Ya-Fang Chen, Ming-Jang Chiu, Ta-Fu Chen, Ping-Huan Tsai, Jen-Hau Chen, Chung-Jen Yen, Sung-Chun Tang, Shin-Joe Yeh, Yen-Ching Chen

**Affiliations:** 1 Institute of Epidemiology and Preventive Medicine, College of Public Health, National Taiwan University, No. 17, Xu-Zhou Road, Taipei, 10055 Taiwan; 2 Department of Neurology, National Taiwan University Hospital, No. 1, Changde Street, Taipei, 10048 Taiwan; 3 Division of Neurology, Department of Internal Medicine, Far Eastern Memorial Hospital, No. 21, Nanya South Road, Banciao District, New Taipei City, 22060 Taiwan; 4 Department of Medical Imaging, National Taiwan University Hospital, No. 1, Changde Street, Taipei, 10048 Taiwan; 5 Department of Geriatrics and Gerontology, National Taiwan University Hospital, No. 1, Changde Street, Taipei, 10048 Taiwan; 6 Department of Internal Medicine, National Taiwan University Hospital, No. 1, Changde Street, Taipei, 10048 Taiwan; 7 Department of Public Health, National Taiwan University, No. 17 Xu-Zhou Road, Taipei, 10055 Taiwan; 8 Research Center for Genes, Environment and Human Health, College of Public Health, National Taiwan University, No. 17 Xu-Zhou Road, Taipei, 10055 Taiwan

## Abstract

Chronic kidney disease has been linked to cognitive impairment and morphological brain change. However, less is known about the impact of kidney functions on cerebral cortical thickness. This study investigated the relationship between kidney functions and global or lobar cerebral cortical thickness (CTh) in 259 non-demented elderly persons. Forty-three participants (16.7%) had kidney dysfunction, which was defined as either a glomerular filtration rate (GFR) of <60 ml/min/1.73 m^2^ or presence of proteinuria. Kidney dysfunction was associated with lower global (*β* = −0.05, 95*%* CI = −0.08 to −0.01) as well as frontal, parietal, temporal, occipital, and insular lobar CTh. In the stratified analysis, the associations were more pronounced in women, *APOE*ε4 non-carriers, and participants with a lower cognitive score. Besides, kidney dysfunction significantly increased the risk of cortical thinning, defined as being the lowest CTh tertile, in the insular lobe (adjusted odds ratio = 2.74, 95% CI = 1.31−5.74). Our results suggested that kidney dysfunction should be closely monitored and managed in elderly population to prevent neurodegeneration.

## Introduction

The worldwide incidence and prevalence of dementia is increasing rapidly in persons of advanced age, which results in a huge global disease burden^1^. Currently, it is estimated that 46.8 million people worldwide are living with dementia, although in some developed countries the prevalence is gradually lowering possibly due to higher education levels and better control of cardiovascular risk factors^2, 3^. Long before clinically evident dementia-related functional impairment, generalized brain atrophy progresses gradually and asymptomatically with age, beginning at middle age^4^. Therefore, it is important to identify potentially preventable factors related to brain atrophy during the subclinical phase. Consequently, the structural neuroimaging, among various potential biomarkers for dementia, serves most closely temporal relationship with the onset of clinically detectable cognitive impairment^5^. The most commonly applied structural neuroimaging technique is magnetic resonance imaging (MRI). In various MRI imaging sequences, physicians can use three-dimensional T1-weighted images that clearly delineate the volume, shape, and thickness of the respective cerebral cortex. Specifically, the volume of cortical regions is a composite measure related to both thickness and surface area. In these two morphometric parameters, cortical thickness more closely reflects pathological changes related to dementia, such as laminar thinning and neuronal loss, while volume loss is largely the result of a reduction in surface area during normal aging^6^.

Several factors have been linked to dementia, such as age, sex, education, apolipoprotein E (*APOE*) ε4 status, and various cardiovascular risk factors such as hypertension, diabetes mellitus, metabolic syndrome, and stroke^7^. In recent years, brain-kidney interaction is increasingly emphasized. Chronic kidney disease (CKD) and dementia are both common diseases in the elderly, and CKD has been proposed as an independent risk factor for cognitive impairment^8^. A population-based study had demonstrated that cognitive impairment is common not only in hemodialysis patients, but also in the less severe stages of CKD^9^. CKD was also linked to subclinical brain MRI abnormalities, such as cerebral small vessel disease, white matter hyperintensities, or global brain atrophy^10–13^. However, less is known about the relationship between kidney functions and cortical thickness of the brain, especially in a nondisabled elderly population. Therefore, the present study aimed to investigate the potential impact of kidney dysfunctions on cerebral cortical thickness in a group of independently living older persons. This study further explored whether age, gender, *APOE*ε4 status and cognitive function affect the association between kidney function and cerebral cortical thickness.

## Methods

### Study Population

This is a cross-sectional study from a prospective cohort study (2011-present). Participants aged 65 years or older were recruited from the annual Elderly Health Checkup (EHC) at National Taiwan University Hospital (NTUH) from 2011 to 2013, and all participants underwent a brain MRI examination, except those with contraindications (e.g., implantation of a cardiac pacemaker, defibrillators or other electronic devices, received intracranial metal coils, or claustrophobia). A total of 397 participants who underwent a brain MRI examination were recruited. To clarify the cross-sectional relationship between kidney function and structural brain change, the MRI examination had to be performed within 6 months of specimen collection, along with questionnaire administration, and other clinical examinations (n = 263). As the study aimed to investigate the brain structural change among elderly people without known destructive or degenerative neurological diseases, we excluded those with a history of stroke (n = 3) or using medication for Alzheimer’s disease (AD, n = 1). After exclusion, 259 participants were included for statistical analysis. Informed consent was obtained from each participant before administration of questionnaires, performing clinical assessments, and collection of biological specimen at NTUH. The research plan, informed consent, questionnaires, and application forms were approved by the Institutional Review Board at NTUH. All methods were carried out in accordance with the approved guidelines and regulations.

### Acquisition of MRI

Brain MRI was performed using a single 1.5-T scanner with high-resolution T1-weighted volumetric MRI scans. All MRI images were processed using FreeSurfer (http://surfer.nmr.mgh.harvard.edu). The FreeSurfer image analysis suite can perform surface extraction, cortical parcellations and thickness computation^14^. The cortical thickness (CTh) of each participant was derived from a surface-based pipeline consisting of several stages, including but not limited to the following: image registration via affine transformation to Montreal Neurological Institute atlas, correction of intensity inhomogeneities, removal of skull using a deformable template model, classification of voxels into white matter and non-white matter based on intensity, tessellation of gray-white matter boundaries, automated correction of topological defects, and surface reconstruction to delineate the gray matter-cerebrospinal fluid and gray matter-white matter borders. Ultimately, cortical thickness was obtained at each vertex by computing the shortest distance between the above two surfaces (gray matter-cerebrospinal fluid and gray-white matter). In the FreeSurfer analysis suite, there were 34 predefined regions of interest in each cerebral hemisphere^15^. Cortical thickness (CTh) was expressed in micrometers as global (whole brain) average and lobar (left and right thickness averaged for frontal, parietal, temporal and occipital lobes) average. The method of cortical thickness measurements has been validated with histological data^16^ and manual measurements^4^, and has been used in several previous studies^6, 17, 18^. Further, total intracranial volume was quantified by automatic segmentation with the FreeSurfer analysis suite. White matter lesions, defined as areas in cerebral white matter that appear abnormally hypointense on T1-weighted or hyperintense on T2-weighted images, were semi-quantitatively graded according to an age-related white matter change rating scale (score 0–3, with a higher score representing more severe lesions) by an experienced neuroradiologist who was unaware of the study population^19, 20^. Lacunes were defined as small lesions (≤15 mm in diameter) with a low signal on T1-weighted and high signal on T2-weighted images. The presence or absence of lacunes was recorded. In addition to the use of continuous CTh variable for analysis, CTh was further organized into tertiles to identify potential susceptible population from a public health perspective. Cerebral cortical thinning was defined as the lowest tertile (T1), whilenormal CTh was defined as the remaining tertiles (T2 + T3).

### Kidney function

To estimate kidney function parameters, the serum creatinine level was obtained from a blood test, and glomerular filtration rate (GFR) was calculated using the Chronic Kidney Disease Epidemiology Collaboration (CKD-EPI) equation^21^:

$$GFR=141\times \,{\rm{\min }}\,{(\frac{Scr}{\kappa },1)}^{\alpha }\times \,{\rm{\max }}\,{(\frac{Scr}{\kappa })}^{-1.209}\times {0.993}^{age}\times 1.018\,(if\,female)$$

In this equation, Scr is serum creatinine (mg/dL), κ is 0.7 for females and 0.9 for males, α is −0.329 for females and −0.411 for males, min indicates the minimum of Scr/κ or 1, and max indicates the maximum of Scr/κ or 1. This equation was more accurate, less biased, and better for precision than the traditionally used Modification of Diet in Renal Disease Study (MDRD) equation^21^. Urine was also collected from each participant for urinalysis. The level of urinary protein was examined using a urine dipstick and was classified into absence (dipstick reading of − or ± ) or presence (1+ to 4+) of proteinuria, with 1 + approximately corresponding to a proteinuria level of 30 mg/dL,which is the cut-off value for microalbuminuria^22, 23^. In this study, ‘kidney dysfunction’ was defined if a participant had either a GFR < 60 ml/min/1.73 m^2^ or presence (≥1+) of proteinuria.

### Covariates

A set of relevant covariates was obtained during the study period (2011–2013). Demographic data included age, gender, body mass index (BMI), years of education, and history of smoking. Medical comorbidities such as hypertension, diabetes mellitus, dyslipidemia, metabolic syndrome, or depressive symptoms were documented based on self-report by the participants, available medical records or laboratory data. Laboratory data from blood specimens included total cholesterol, triglycerides, glucose, and *APOE*ε4 status. Cognitive function was evaluated by a trained assistant using the Taiwan version of Montreal Cognitive Assessment (MoCA-T)^24^, which is a brief test that evaluates several cognitive domains, including visuospatial, executive function, language, verbal memory, attention, and orientation. The MoCA-T scores range from 0 to 30. Participants with MoCA-T score of less than 24 would be classified as having cognitive impairment^24^. In addition, participants were surveyed using the self-report Center for Epidemiologic Studies Depression (CES-D) scale to assess symptoms of depression, with 20-item score that ranged from 0 to 60. Participants with scores 16 or higher on CES-D scale would be assigned as presence of depressive symptoms^25^.

### Statistical analysis

To examine differences in characteristics between participants with or without kidney dysfunction, a Chi-square test was used for categorical variables, and Student’s *t* test or Mann-Whitney *U*-test were used for continuous variables. Correlation between GFR and CTh (global and lobar) was evaluated by Spearman’s rank sum test, and a partial correlation model (represented as *ρ*) was applied after adjustment of age, gender, years of education, MoCA-T score, and *APOE*ε4 status.

To explore whether the level of GFR or presence of kidney dysfunction were related to global and lobar CTh, multivariable linear regression models were applied. Because one single GFR unit was relatively small, it was expressed as every 10 ml/min/1.73 m^2^ increase in the regression models. In model 1, important covariates including age, gender, years of education, MoCA-T score, *APOE*ε4 status, and intracranial volume were adjusted. In model 2, degree of white matter lesion, presence of lacunes, BMI, hypertension, diabetes mellitus, and smoking were added to test whether kidney dysfunction related to cortical thinning was independent of cerebral small vessel disease and vascular risk factors. A logistic regression model was used to estimate adjusted odds ratio (aOR) and 95% confidence intervals (CI) with cortical thinning versus normal CTh for GFR values or kidney dysfunction, respectively. The adjusted covariates were the same as the linear regression models described above. False discovery rate correction was used to correct for inflated type I error because of multiple comparisons. An additional stratified analysis was performed based on age groups (65–74 *vs* ≥75 years old), gender, cognitive status (MoCA-T < 24 *vs* MoCA-T $\ge$ 24), and *APOE*ε4 status (carriers *vs* non-carriers). All analyses were performed using SAS version 9.3 (SAS Institute Inc, Cary, NC, USA). All statistical tests were two-sided, and *P* values of <0.05 were considered significant.

## Results

### Characteristics of study population

A total of 259 participants (mean age 73.0 ± 5.2 years old, male 45.6%) from an elderly health checkup program who underwent a comprehensive baseline data collection and brain MRI study were included for analysis. The mean global thickness of the participants was 2.24 ± 0.12 mm. Thirty-five participants had a GFR of <60 ml/min/1.73 m^2^, thirteen had presence of proteinuria, and a total of 43 participants were classified as having kidney dysfunction. For those with kidney dysfunction, they were generally older, were less educated, had a lower MoCA-T score, had more severe white matter lesions, had thinner global and most of the lobar CTh except the limbic lobe (Table 1). The GFR values were positively correlated with the global and lobar CTh in an unadjusted model (Spearman’s *r*, 0.16–0.31; all *P* < 0.05), but partial correlation existed in only the frontal (*ρ* = 0.16, *P* = 0.01) and insular (*ρ* = 0.14, *P* = 0.03) lobe after a partial adjustment of age, gender, years of educational, MoCA-T score, and *APOE*ε4 status.

**Table 1:** Characteristics of the study participants according to the status of kidney dysfunction.

	Kidney Dysfunction		
	Yes (n = 43)	No (n = 216)	*P* value^b^
Age $\mbox{--}$ year	76.4 ± 5.9	72.3 ± 4.8	<0.001
Male gender $\mbox{--}$ no. (%)	26 (60.5%)	92 (42.6%)	0.03
*APOE* ε4carriers $\mbox{--}$ no. (%)	5 (11.6%)	37 (17.3%)	0.36
Years of education	11.5 ± 4.8	13.7 ± 3.5	0.08
Body mass index (kg/m^2^)	24.9 ± 3.5	23.9 ± 3.0	0.09
Metabolic syndrome $\mbox{--}$ no. (%)	24 (58.5%)	80 (38.5%)	0.02
Hypertension $\mbox{--}$ no. (%)	35 (81.4%)	145 (67.4%)	0.07
Diabetes mellitus $\mbox{--}$ no. (%)	11 (25.6%)	30 (13.9%)	0.06
Dyslipidemia $\mbox{--}$ no. (%)	29 (67.4%)	124 (57.4%)	0.22
Ever smoking $\mbox{--}$ no. (%)	24 (11.1%)	10 (23.3%)	0.03
Depressive symptoms $\mbox{--}$ no. (%)^a^	7 (16.3%)	20 (9.3%)	0.17
MoCA-T score	24.6 ± 0.62	26.6 ± 0.18	0.01
GFR -ml/min/1.73 m^2^	55.5 ± 17.3	80.4 ± 10.3	<0.001
Degree of white matter lesions	1.9 ± 0.8	1.6 ± 0.9	0.01
Presence of lacunes – no. (%)	6 (14.0%)	20 (9.3%)	0.35
Global CTh - mm	2.17 ± 0.11	2.26 ± 0.11	<0.001
Frontal CTh - mm	2.31 ± 0.11	2.39 ± 0.12	<0.001
Temporal CTh - mm	2.56 ± 0.17	2.66 ± 0.15	<0.001
Parietal CTh - mm	1.93 ± 0.10	2.02 ± 0.12	<0.001
Occipital CTh - mm	1.64 ± 0.07	1.70 ± 0.09	<0.001
Limbic CTh - mm	2.46 ± 0.14	2.50 ± 0.15	0.10
Insular CTh - mm	2.72 ± 0.17	2.84 ± 0.16	<0.001

Data are presented as mean ± standard deviation. ^a^Depressive symptomsare defined as scores 16 or higher on Center for Epidemiologic Studies Depression (CES-D) Scale, a self-report scale to assess symptoms of depression with 20-item score ranged from 0 to 60. ^b^ *P* values were obtained from Student’s *t* tests (normally-distributed continuous variables), Mann-Whitney *U* tests (non-normally distributed continuous variables), and Chi-square tests (categorical variables) for comparing participants with or without kidney dysfunction.

### Linear association between CTh and kidney function (GFR or kidney dysfunction)

In multiple linear regression models (Table 2), every 10–ml/min/1.73 m^2^ increase in GFR was significantly associated with a greater CTh for the global (*β* = 0.01, 95% CI = 0.0005–0.02), frontal (*β* = 0.01, 95% CI = 0.002–0.02), and insular lobes (*β* = 0.02, 95% CI = 0.003–0.03) if model 1 was applied; however, the association between GFR and global CTh did not exist if model 2 was applied. Nevertheless, the presence of kidney dysfunction was independently associated with a lower global CTh (*β* = −0.05, 95% CI = −0.08–−0.01, *P* = 0.01), as well as frontal, parietal, temporal, occipital and insular lobe CTh (*β* = −0.04 ~ −0.11; Table 2). The results were similar when model 2 was applied except temporal and limbic lobe. The significant findings remained after correcting multiple tests. In terms of a lower global CTh, kidney dysfunction had similar impacts with 6.5 years of aging (estimated by dividing the coefficient for kidney dysfunction [*β* = −0.0468] by the coefficient for age per year [*β* = −0.0072]).

**Table 2:** Association between kidney function and CTh by multiple linear regression models.

CTh (mm)	GFR (every 10 ml/min/1.73 m^2^)				Kidney Dysfunction^a^			
	Model 1^b^		Model 2^b^		Model 1^b^		Model 2^b^	
	*β* (95% CI)	*P* value	*β* (95% CI)	*P* value	*β* (95% CI)	*P* value	*β* (95% CI)	*P* value
Global	**0**.**01** (**0**.**00005**, **0**.**02**)	**0**.**05**	0.01 (−0.0005, 0.02)	0.06	**−0**.**05** (**−0**.**08**, **−0**.**01**)	**0**.**01** ^c^	**−0**.**04** (**−0**.**08**, **−0**.**01**)	**0**.**02** ^c^
Frontal lobe	**0**.**01** (**0**.**002**, **0**.**02**)	**0**.**02**	**0**.**01** (**0**.**002**, **0**.**02**)	**0**.**02**	**−0**.**05** (**−0**.**09**, **−0**.**01**)	**0**.**02** ^c^	**−0**.**05** (**−0**.**09**, **−0**.**01**)	**0**.**02** ^c^
Temporal lobe	0.009 (**−**0.005, 0.02)	0.23	0.007 (**−**0.007, 0.02)	0.34	**−0**.**06** (**−0**.**11**, **−0**.**01**)	**0**.**03** ^c^	**−**0.05 (**−**0.10, 0.002)	0.06
Parietal lobe	0.01 (**−**0.0004, 0.002)	0.06	0.01 (**−**0.0007, 0.02)	0.07	**−0**.**05** (**−0**.**09**, **−0**.**01**)	**0**.**03** ^c^	**−0**.**04** (**−0**.**08**, **−0**.**003**)	**0**.**04** ^c^
Occipital lobe	0.006 (**−**0.003, 0.001)	0.20	0.006 (**−**0.003, 0.01)	0.18	**−0**.**04** (**−0**.**07**, **−0**.**01**)	**0**.**01** ^c^	**−0**.**04** (**−0**.**07**, **−0**.**01**)	**0**.**01** ^c^
Limbic lobe	0.005 (**−**0.008, 0.02)	0.46	0.004 (**−**0.009, 0.02)	0.58	**−**0.02 (**−**0.07, 0.03)	0.44	**−**0.01 (**−**0.06, 0.04)	0.66
Insular lobe	**0**.**02** (**0**.**003**, **0**.**03**)	**0**.**02**	**0**.**02** (**0**.**001**, **0**.**03**)	**0**.**04**	**−0**.**11** (**−0**.**17**, **−0**.**05**)	<**0**.**001** ^c^	**−0**.**09** (**−0**.**15**, **−0**.**04**)	**0**.**002** ^c^

^a^Kidney dysfunction is defined as either GFR < 60 ml/min/1.73 m^2^ or presence (≥1+) of proteinuria. ^b^Model 1 was adjusted for age, gender, education years, *APOE* ε4 carrier status, MoCA-T score, and intracranial volume; Model 2 was additionally adjusted for degree of white matter lesion, lacunes, hypertension, diabetes mellitus, body mass index, and smoking. ^c^False discovery rate *q* value < 0.05. Numbers in bold indicated significant findings (before correction for multiple comparisons).

### Association between kidney function (GFR or kidney dysfunction) and cortical thinning

No significant association was observed between GFR and global or lobar cortical thinning in the logistic regression models (Table 3), except borderline finding in the frontal lobe (aOR = 0.74, 95% CI = 0.66–0.99, *P* = 0.04) which was attenuated after correction for multiple comparisons. On the other hand, the presence of kidney dysfunction was associated with an increased risk of insular lobar cortical thinning (aOR = 2.74, 95% CI = 1.31–5.74, *P* = 0.01; Table 3). However, the effect did not exist for global or other lobar cortical thinning.

**Table 3:** Association of kidney function and cortical thinning (lower tertile of global or lobar CTh) by logistic regression models.

Cortical thinning	GFR (every 10 ml/min/1.73 m^2^)		Kidney Dysfunction^a^	
	aOR (95% CI)^b^	*P* value	aOR (95% CI)^b^	*P* value
Global	0.91 (0.73–1.14)	0.42	1.82 (0.85–3.91)	0.13
Frontal lobe	**0**.**79** (**0**.**63–0**.**99**)	**0**.**04**	1.83 (0.84–3.98)	0.13
Temporal lobe	0.92 (0.74–1.13)	0.41	1.57 (0.74–3.33)	0.24
Parietal lobe	0.95 (0.76–1.18)	0.63	1.33 (0.62–2.87)	0.47
Occipital lobe	0.96 (0.78–1.19)	0.71	1.96 (0.93–4.16)	0.08
Limbic lobe	0.93 (0.76–1.16)	0.54	0.84 (0.39–1.82)	0.66
Insular lobe	0.86 (0.70–1.05)	0.14	**2**.**74** (**1**.**31–5**.**74**)	**0**.**01** ^c^

^a^Kidney dysfunction is defined as either GFR < 60 ml/min/1.73 m^2^ or presence (≥1+) of proteinuria. ^b^The model was adjusted for age, gender, education years, *APOE* ε4carrier status, MoCA-T score, and intracranial volume. ^c^False discovery rate q value was 0.056. Numbers in bold indicated significant findings.

### Stratified analyses by important potential confounders

No significant interaction existed between kidney dysfunction and age, gender, cognitive or *APOE*ε4 status for global CTh (*P*_interaction_ = 0.98, 0.08, 0.54, and 0.06, respectively). If using the linear regression model 1, GFR had a significant impact on global CTh in those participants with cognitive impairment (MoCA-T < 24) (*β* = 0.02, 95% CI = 0.002–0.04; Table 4). In contrast, kidney dysfunction was independently associated with lower global CTh in subgroups of younger age (aged 65–74 years-old, *β* = −0.05, 95% CI = −0.10 to −0.01), female (*β* = −0.09, 95% CI = −0.15 to −0.04), and *APOE*ε4 non-carriers (*β* = −0.06, 95% CI = −0.10 to −0.02; Table 4). The impact of kidney dysfunction on women was equivalent to 20.5 years of aging, while the impact on *APOE* ε4 non-carriers was equivalent to 6.7 years of aging.

**Table 4:** Association of kidney function and global CTh by multiple linear regression models, stratification by important factors.

Subgroup		N	GFR (every 10 ml/min/1.73 m^2^)		Kidney Dysfunction^a^	
			*β* (95% CI)^b^	*P* value	*β* (95% CI)^b^	*P* value
Age	$\ge$75	84	0.02 (**−**0.003, 0.04)	0.09	**−**0.06 (**−**0.13, 0.007)	0.08
	<75	175	0.01 (**−**0.002, 0.02)	0.10	**−0**.**05** (**−0**.**10**, **−0**.**007**)	**0**.**02**
Gender	Male	118	0.007 (**−**0.008, 0.02)	0.37	**−**0.02 (**−**0.07, 0.03)	0.51
	Female	141	0.02 (**−**0.0001, 0.03)	0.05	**−0**.**09** (**−0**.**15**, **−0**.**04**)	**0**.**002**
*APOE*ε4	Carrier	42	0.01 (**−**0.02, 0.04)	0.42	0.005 (**−**0.10, 0.11)	0.92
	Non**−**carrier	215	0.01 (**−**0.001, 0.02)	0.09	**−0**.**06** (**−0**.**10**, **−0**.**02**)	**0**.**01**
MoCA**−**T	$\ge$24	213	0.006 (**−**0.006, 0.02)	0.32	**−**0.04 (**−**0.08, 0.007)	0.19
	<24	46	**0**.**02** (**0**.**002**, **0**.**04**)	**0**.**03**	**−**0.07 (**−**0.15, 0.006)	0.07

^a^Kidney dysfunction is defined as either GFR < 60 ml/min/1.73 m^2^ or presence (≥1+) of proteinuria. ^b^The models were routinely adjusted for factors including age, gender, education years, *APOE* ε4 carrier status, MoCA-T score, and intracranial volume, except the factors used for stratifications. Numbers in bold indicated significant findings.

## Discussion

The present study demonstrated that decreased kidney function was associated with cerebral cortical thinning in an independently living elderly population. The association was more salient and consistent when combining a lower GFR and proteinuria within the kidney dysfunction population, rather than focusing on GFR alone. Traditionally, a lower GFR was viewed as a hallmark of CKD. Nevertheless, proteinuria was increasingly being viewed as another important indicator and a screening tool for CKD^26^. Proteinuria revealed dysfunction of the glomerular barrier and often preceded any detectable decline in renal filtration function^26^. In fact, current guideline of diagnosing CKD required the presence of markers of kidney damage (such as proteinuria) or decreased GFR for more than 3 months^23^. As our study had a cross-sectional design, we were unable to diagnose participants with CKD but could only label them as having “kidney dysfunction”. Nonetheless, our findings implied that both glomerular filtration rate and barrier dysfunction may contribute to the morphological changes in the cerebral cortex.

Previous studies usually investigated the impact of reduced kidney function on the whole brain volume. For example, in a study including 1253 community-dwelling elders without dementia, a low GFR or the presence of microalbuminuria was significantly associated with generalized brain atrophy^12^. A Japanese study on 610 adults recruited from health examination confirmed that a lower GFR and the presence of CKD were both associated with generalized cerebral atrophy^13^. One study in the Netherlands, however, did not demonstrate a significant association between GFR and total gray matter volume in 434 non-disabled elderly persons^10^.

There were only a few studies that explored the relationship between kidney function and cortical thickness. A Korean study investigated the regional cortical thickness among 162 patients clinically diagnosed with AD, and the results indicated that patients in the lowest GFR quartile had a significantly thinner cortex than those in the highest quartile, especially in the temporal and parietal lobes^27^. A recently published study from the United States examined the associations between various systemic markers of disease and cortical thickness in 138 healthy middle-aged and elder adults^28^. They found that kidney function was related to cortical thickness in widespread association and sensory-motor areas. Our findings paralleled their results, however, there were several differences between the two studies that should be emphasized. First, we included not only the glomerular filtration rate but also proteinuria, a potent marker for early kidney damage, compared to their use of blood markers such as GFR, creatinine, and BUN to represent kidney function. Besides, their study employed the surface-based general linear model analysis which incorporated covariate once a time, while we applied a multivariable linear regression to embrace all potential confounders. In doing so, common confounding factors, such as age, sex, educational level, *APOE*ε4 carrier status, and cognitive function could be incorporated into the model, in order to examine these associations in a more comprehensive method. In summary, both studies addressed an important viewpoint that kidney function was closely related to brain morphology even among healthy individuals.

**Figure 1:**
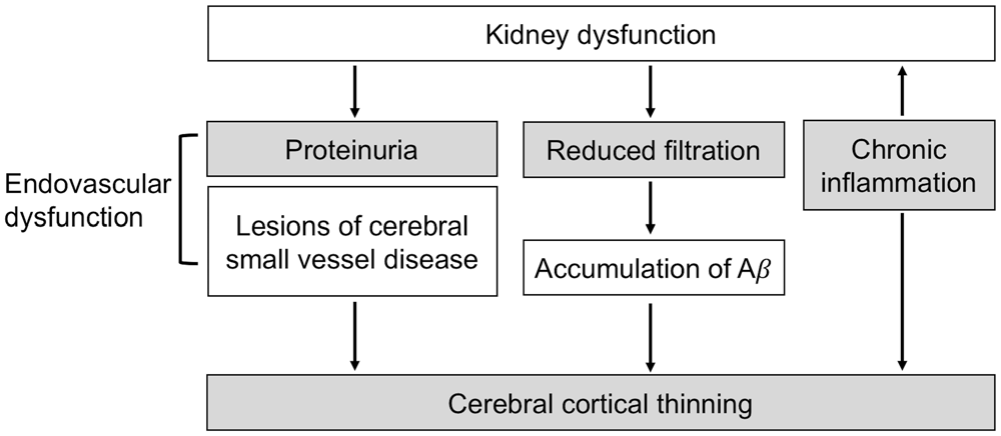
Postulated mechanism of kidney dysfunction in relation to cortical thinning.

The reasons that kidney dysfunction had a negative effect on the cortical thickness were probably multifactorial (Fig. 1). It had been observed that systemic catabolism of amyloid beta was largely performed by kidney or liver in mice^29^. In both probable AD patients and normal controls, the serum creatinine level was significantly correlated with plasma amyloid beta (either A *β* 40 or A *β* 42)^30^. Consequently, the accumulation of A *β* in the body and its downstream neurodegeneration was still the main hypothetical model for the pathophysiological process in AD^5^. Therefore, reduced kidney function could lead to impaired peripheral excretion and an abnormal accumulation of circulating A *β*, ultimately resulting in cortical thinning.

Another explanation might be related to a background atherosclerotic state and cerebrovascular dysregulation. Many studies have found that CKD independently contributed to subclinical brain lesions on MRI, such as silent brain infarcts, lacunes, white matter hyperintensities, or cerebral microbleeds^10, 11, 31–35^. These were mainly lesions of cerebral small vessel disease and could lead to brain atrophy and cortical thinning^36^. In our study, the independent association between GFR and cortical thickness was attenuated after adjustment of white matter lesions, lacunes, and other vascular risk factors (model 2), but the association persisted between kidney dysfunction and cortical thickness. These results implied that both an excretion abnormality (GFR *per se*) and endovascular dysfunction (proteinuria) were associated with cortical thinning, and the underlying pathophysiological process may be independent of cerebral small vessel disease.

Yet another mechanism may contribute to the chronic inflammatory state that exists in patients with CKD^37^. It has been shown that inflammation could mediate the relationship between metabolic syndrome and cortical thinning^38^. Additional work to explore the contribution of inflammatory markers in the kidney dysfunction related cortical thinning is worthy of investigation.

One interesting finding was that kidney dysfunction was associated with profound cortical thinning in the insular lobe. Although the function of the insula was not fully understood, it was usually linked to emotional process, autonomic function and interoception^39, 40^. Abnormal changes in insular cortical thickness have been found in patients with chronic musculoskeletal pain, abdominal pain, migraine or smoking addiction^39, 41–43^. In patients with mild AD, depression and anxiety symptoms were found to be associated with thinning of the insular cortex^44^. We did not find a significant correlation between insular cortical thickness and depressive symptoms scores (based on Center for Epidemiological Studies Depression scale) in our study population. Possible factors contributing to the kidney-dysfunction related insular thinning included circulating toxin, accumulation of A *β*, or oxidative stress brought by endothelial dysfunction.

Several demographic factors have been found to be associated with changes in cortical thickness. Increasing age was generally associated with less cortical thickness^4, 45, 46^. In our subgroup analysis, we found that in the young-old group (65–74 years-old), kidney dysfunction was significantly related to a lower global CTh, while the old-old group ($\ge$75 years-old) also demonstrated a non-significant relationship. Hence, increasing age did not seem to have an important effect on kidney-dysfunction related cortical thinning. However, close monitoring of kidney function during earlier lifespan was important since the accumulation of A*β* could start from middle age^5^. Secondly, women tended to have a greater cortical thickness than their age-matched counterpart^45, 46^, and our results were in line with these findings (women *vs* men: + 47.7 μm, 95% CI = 21.4–74.1 μm in age-adjusted global cortical thickness). Interestingly, we found that kidney dysfunction had more prominent effects on global CTh in women (equivalent to an aging effect of 20.5 years). Whether this was because men had more competing risk factors of neurodegeneration, or women were more susceptible to kidney dysfunction, is worthy of further exploration.

Generally, *APOE*ε4 carriers, even those with normal cognition, had lower cortical thickness^17, 47^. We found that *APOE*ε4 status demonstrated a borderline modification effect (*P*_interaction_ = 0.06) on the association between kidney dysfunction and global CTh, and *APOE*ε4 non-carriers were more likely to be affected by kidney dysfunction. A recent study that demonstrated the association between kidney function factor and CTh did not find *APOE*ε4 status being an effect modifier^28^. However, their finding was in fact concordant with ours that the negative impact of kidney function on the CTh was more prominent among *APOE*ε4 non-carriers. The results suggested that kidney function should be carefully assessed and monitored in *APOE*ε4 non-carriers who have a risk of neurodegeneration. Further, the association between higher GFR and greater global CTh was found only in participants with cognitive impairment (MoCA-T < 24). Interestingly, participants with cognitive impairment did not have lower CTh than those without (2.23 ± 0.12 mm *vs* 2.24 ± 0.12 mm, respectively; *P* = 0.61). It could be speculated that in those who already have cognitive impairment, worsening systemic factors such as a reduction in GFR would have a greater effect on brain morphology.

The present study had some limitations. First, this study was designed to be cross-sectional, and hence, the causality of kidney function and cortical thinning could not be directly inferred. Second, we used urinary dipstick as a semi-quantitative analysis for proteinuria. A more precise method would have been the quantitative measurement of the urinary albumin-to-creatinine ratio to determine the degree of albuminuria. That said, a dipstick test result of less than 1+ usually had a high negative predictive value with a minimal risk of missed diagnosis of microalbuminuria^48^. Additional study to explore a possible linear relationship between the level of albuminuria and CTh is desirable. Third, we did not use a commonly used case-control method to compare differences in regional CTh^49–51^, as we studied a relatively homogeneous group of participants without any known neurodegenerative diseases or significant medical illness. Moreover, we focused on a potential preclinical association between kidney function changes and CTh. We did not perform the surface-based analysis to generate maps of regional differences by the Freesurfer suite like some researchers did^28^, since we applied a multivariable linear regression approach to embrace all potential confounders into one model. Lastly, this study was conducted among Taiwanese elderly adults, who were mainly Han Chinese, so it was limited to generalize our study results to other racial or ethnic groups.

In summary, our findings suggest that kidney dysfunction is associated with cerebral cortical thinning in community-dwelling, non-demented elderly population. Further prospective study is warranted to evaluate whether interventions to protect kidney function or to reduce proteinuria could effectively slow the progression of cerebral cortical thinning. Thus, the critical role of kidney function can be better elucidated in the process of neurodegeneration.
